# Reversing liver sinusoidal endothelial aging restores clearance of amyloid-beta and LDL

**DOI:** 10.1016/j.jbc.2026.113608

**Published:** 2026-09-26

**Authors:** Sam J. de Leve, Xiangdong Wang, Helia Alavifard, Lily Dara, Laurie D. DeLeve

**Affiliations:** Division of Gastrointestinal and Liver Diseases, Keck School of Medicine, Los Angeles, California, USA

**Keywords:** Alzheimer disease, cell-cell interaction, cyclic GMP (cGMP), dyslipidemia, endocytosis, lipoprotein metabolism, soluble guanylate cyclase, capillarization, fenestration, heparin-binding epidermal growth factor-like growth factor

## Abstract

In healthy liver, large macromolecules pass freely into the space of Disse through liver sinusoidal endothelial cell (LSEC) fenestration. In aging, LSEC fenestration is largely lost (pseudocapillarization). We hypothesized that restoring aged LSECs’ fenestration would enhance passage into the space of Disse of large-diameter (>50 nm) triglyceride-rich lipoproteins to which the majority of circulating amyloid-beta is bound and permit hepatocyte uptake of amyloid-beta: in aged rats, 2 weeks of the soluble guanylate cyclase activator cinaciguat restored fenestration; circulating amyloid-beta 40 levels halved, returning to levels found in young rats. Surprisingly, cinaciguat also reversed age-related increases in LDL (a smaller, 20–30 nm diameter lipoprotein), which crosses the sinusoidal endothelial barrier even when LSECs are defenestrated. We therefore investigated additional mechanisms by which LSECs promote hepatocyte clearance. Whereas primary hepatocytes had minimal uptake of amyloid-beta 40 or LDL, hepatocytes cultured in young rat LSEC conditioned medium retained their uptake function. Aged rat LSEC conditioned medium did not facilitate hepatocyte amyloid-beta 40 and LDL uptake but treating aged rats with cinaciguat restored aged rat LSECs paracrine signaling. We discovered that heparin-binding epidermal growth factor-like growth factor (HB-EGF) mediates this LSEC-hepatocyte interaction and that HB-EGF is synthesized but not secreted by aged rat LSECs; cinaciguat treatment restores aged LSECs’ HB-EGF secretion. Treatment reversed age-associated loss of LDLR and low-density lipoprotein receptor-related protein-1 surface expression in rat hepatocytes. This study identifies a candidate therapy to address age-related increases in circulating amyloid-beta and LDL and elucidates an intercellular crosstalk mechanism that governs these phenomena.

In the development of Alzheimer's disease, amyloid-beta (Aβ) plaques do not only derive from Aβ produced in the brain, but also from Aβ in the circulation that crosses the blood-brain barrier (1). Likewise, peripheral organs play a significant role in clearing circulating Aβ, with the liver clearing the majority of injected Aβ (60%) in young, healthy animals (2); when peripheral clearance declines, both peripheral and brain Aβ increase, demonstrating interdependence between peripheral and central nervous system Aβ levels 3, 4, 5, 6, 7, 8. Studies highlight low-density lipoprotein receptor-related protein-1 (LRP1) as the principal hepatic receptor responsible for Aβ uptake and were associated with age-related decreases in hepatic LRP1 expression and reduced Aβ clearance (6). Because hepatic LRP1 regulation is not well understood, identifying its role in Aβ clearance does not, in itself, suggest a therapeutic approach.

In addition to its role in clearing Aβ, the liver is also the principal organ responsible for lipoprotein metabolism, leading us to investigate the possibility of enhancing Aβ clearance by increasing clearance of the lipoproteins to which Aβ is bound. Ninety percent of plasma Aβ40 and 97% of Aβ42 are bound to lipoproteins in humans in healthy aging (9) with 58% bound to the triglyceride-rich lipoproteins (TRLs)– chylomicrons and VLDL; a further 23% is bound to LDL and 19% to HDL (10). Increasing hepatic uptake of TRLs will increase clearance of ligands bound to them, such as Aβ.

To exit the liver’s microcirculation and reach the hepatocyte for clearance, TRLs such as chylomicron remnants and VLDL must first pass through the liver sinusoidal endothelial cells (LSECs) fenestrae 11, 12. With aging, even healthy aging, the size and number of these fenestrae progressively decline, so-called pseudocapillarization 13, 14, 15, 16 (Supporting Fig. S1). Because chylomicron remnants are sieved through LSEC fenestrae (11) and the diameter of the LSEC fenestrae size-limit which chylomicron remnants can pass through, pseudocapillarization has long been thought to contribute to delayed clearance of postprandial chylomicron remnants in older people (15) and Alzheimer’s disease is associated with greater delays in post-prandial clearance (10). Taken together, these studies suggest that LSEC loss of fenestration with pseudocapillarization will delay clearance of Aβ bound to TRLs.

By contrast, small-diameter lipoprotein fractions like LDL and HDL can enter the space of Disse relatively unobstructed, even in knockout models with near-total absence of LSEC fenestrae that causes gross chylomicronemia (12). Though circulating LDL increases in older people, and these increases are attributed to reduced hepatic clearance (17), impaired hepatic clearance of these smaller lipoprotein particles cannot be attributed to loss of LSEC porosity alone. This suggests a second mechanism for reduced hepatic clearance in aging.

Nevertheless, if LSEC porosity could be restored, it could reverse delays in chylomicron remnant clearance and thereby lower levels of TRL-bound Aβ. Existing literature indicates that the LSECs’ VEGF-nitric oxide (NO) pathway is critical to regulating its permeability, with soluble guanylate cyclase (sGC) as a particularly impactful pivot point in that pathway 18, 19. sGC catalyzes synthesis of cyclic guanosine monophosphate (cGMP). Cyclic GMP is a key second messenger involved in a wide range of intracellular signaling pathways, and because sGC synthesis of cGMP is NO-dependent, reduction in NO bioavailability, as observed in endothelial dysfunction of disease or aging, leads to reduced cGMP generation and, by implication, cGMP-dependent signaling. Conversely, pharmaceutical activation of sGC restores intracellular cGMP levels, with associated restoration of LSEC morphology and function in chronic liver disease; in young rats with thioacetamide-induced cirrhosis, in which LSECs lack fenestration, treatment with an sGC activator restores liver sinusoidal endothelial fenestration and normalizes secretion of paracrine growth factors such as heparin-binding epidermal growth factor-like growth factor (HB-EGF) 18, 20.

These studies investigate the hypothesis that, in response to sGC activation, aged, pseudocapillarized LSECs would return to normal phenotype, and allow for clearance of TRL bound Aβ. In the course of this investigation, we uncovered how LSEC aging impairs hepatocyte clearance of small-diameter particles, and whether treating pseudocapillarized LSECs alleviates that impairment. Collectively, these studies demonstrate that aging negates LSECs’ ability to promote hepatocyte Aβ and LDL uptake, and they identify the molecule that mediates this effect; by restoring aged LSECs’ NO-sGC-cGMP pathway, LSECs’ regain their paracrine signaling that promotes hepatocyte uptake, normalizing age-induced elevations in circulating LDL and Aβ levels.

## Results

### Soluble guanylate cyclase activation restores aged liver sinusoidal endothelial cells to normal phenotype

In order to investigate whether reversing LSEC pseudocapillarization would have a positive effect on circulating lipoproteins and Aβ, we first confirmed our candidate therapy would restore aged LSECs’ morphology and function. A key determinant of LSEC phenotype is the VEGF-nitric oxide-sGC-cyclic guanine nucleotide monophosphate (cGMP) pathway 18, 19. To activate this pathway, aged rats were assigned at random to 2 weeks of daily solvent control intragastrically or sGC activator cinaciguat, 0.3 mg/kg intragastrically. Indeed, despite a 60% reduction in cGMP in LSECs from aged rats compared to young ones (Fig. 1*A*), cinaciguat treatment normalized aged LSECs' cGMP and restored both morphology and function (Fig. 1, *A*–*C*). LSECs from aged, cinaciguat-treated rats were fully fenestrated, just like their young counterparts (Figs 1*B*, Supporting S2). Cinaciguat treatment also substantially enhanced aged LSECs' endocytic capacity using the gold standard test 21, 22, 23: uptake of the Stabilin-1 and -2 ligand FITC-labeled formaldehyde-treated serum albumin (Fig. 1*C*). Together, these data demonstrate that cinaciguat treatment effectively addresses the most-studied elements of age-related LSEC pseudocapillarization.

**Figure 1:**
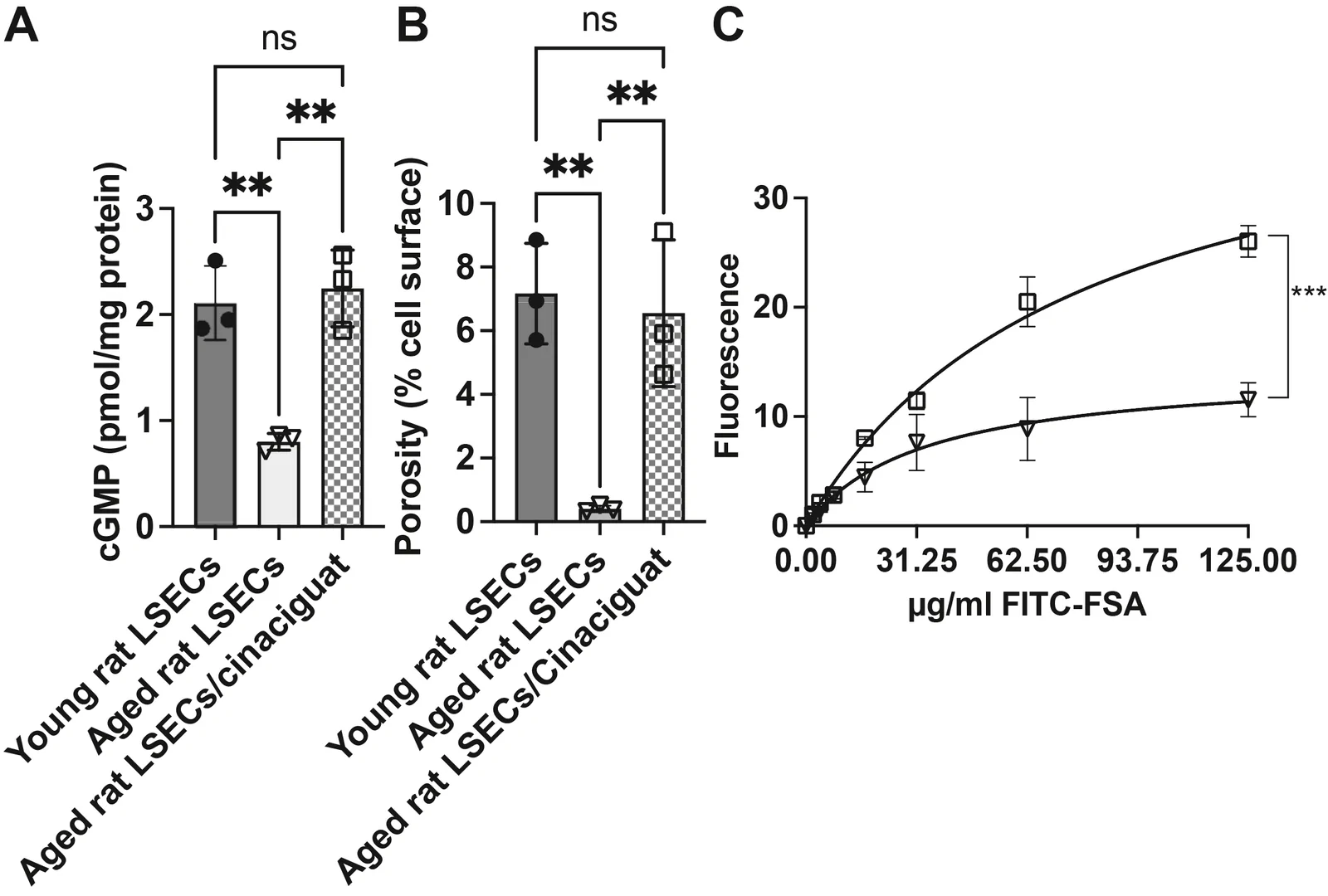
**Effect of sGC activator on LSEC morphology and function in aged rats.***A*, cGMP measured by ELISA in LSEC lysate, (*B*) porosity, percentage of cell surface covered by fenestrae, measured by scanning electron microscopy and quantified by ImageJ. *C*, endocytic capacity for the model stabilin-1,2 ligand FITC-formaldehyde-treated serum albumin, measured over two hours. Young rats (*closed circles*, *dark columns*), aged (*inverted triangles*, *light columns*), and aged cinaciguat-treated rats (*open squares*, *checkered columns*) Figure 1*A*: One-way ANOVA was significant (*p* = 0.0017) with Tukey’s multiple comparison tests as follows: young *versus* aged rat LSEC CM *p* = 0.0038; LSEC CM aged rat ± cinaciguat *p* = 0.0023. Figure 1*B*: One-way ANOVA was significant (*p* = 0.0039) with Tukey’s multiple comparison tests as follows: young *versus* aged rat LSEC CM *p* = 0.0052; LSEC CM aged rat ± cinaciguat *p* = 0.0083. Figure 1*C*: Endocytic capacity was plotted in a saturation-binding plot and assessed by nonlinear regression with best fit by a Michaelis-Menten sigmoidal model; Vmax (maximal internalization rate) was compared between groups. *p* < 0.0001. n = 3 biological replicates. LSEC, liver sinusoidal endothelial cell.

### Cinaciguat treatment normalizes age-related elevation of circulating Aβ-40, native LDL, and oxidized LDL

With loss of LSEC porosity in aging, fewer large diameter macromolecules penetrate into the space of Disse where they can be taken up by hepatocytes during each pass through the liver (15). This reduction in hepatic clearance allows noxious substances more opportunities to wreak havoc in the peripheral and cerebral vasculature, creating a near-perpetual elevation of circulating TRLs with their attendant association with Alzheimer's dementia and atherosclerotic cardiovascular disease risk 10, 11, 24, 25. Intestinally-derived Aβ, and in fact the majority of circulating Aβ, is bound to these particles (26). We hypothesized that restoring LSEC fenestration would increase hepatocyte access to and uptake of these large diameter lipoproteins, thereby facilitating hepatic clearance of intestinally-derived Aβ in the periphery. To examine the impact of age-related loss of fenestration on hepatic clearance and of restoration of fenestration, we compared plasma levels of Aβ-40 in young rats, aged rats, and in rats treated with 2 weeks of cinaciguat. Prior work indicates that the observed dynamics of plasma Aβ40 levels with aging reliably match what one would expect from age-related impairments of hepatic clearance. Moreover, Aβ40 is also the most relevant isoform for studying lipoprotein-bound Aβ40, where Aβ40 makes up 50% of the lipoprotein-bound Aβ isoforms (the remainder being evenly distributed between five other isoforms) (10). These features make Aβ40 the most convincing Aβ isoform to link cinaciguat-induced reversal of liver sinusoidal endothelial (LSEC) pseudocapillarization and circulating Aβ levels.

We found a two-fold increase in Aβ-40 in aged rats and did indeed find that cinaciguat treatment reversed the age-related elevations in Aβ-40, the most prevalent Aβ isoform produced in the periphery and present in the circulation (Fig. 2*A*). These data are consistent with cinaciguat's ability to induce fenestration in aged, pseudocapillarized LSECs, allowing for clearance of TRL-bound Aβ.

**Figure 2:**
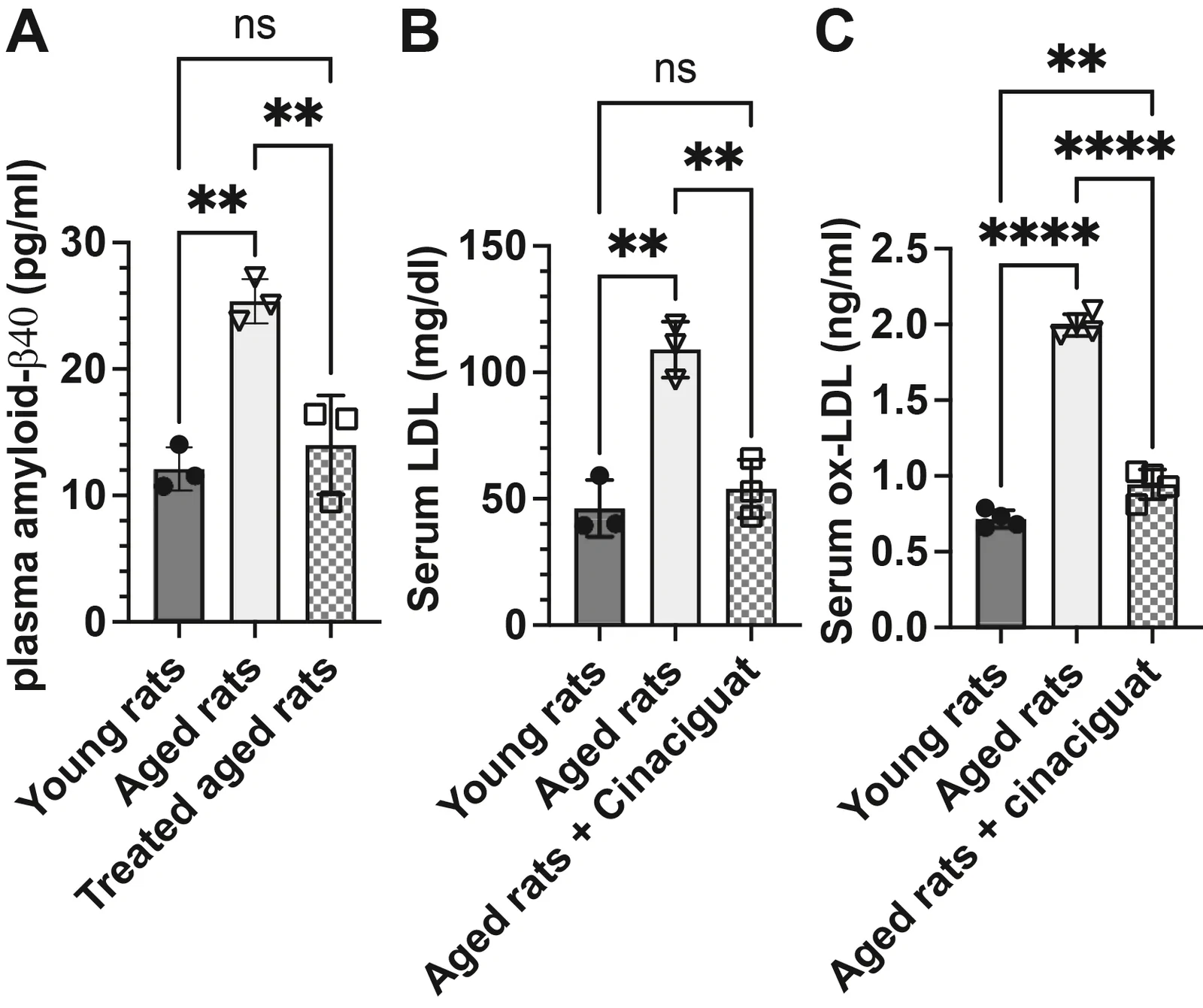
**Effect of sGC activator cinaciguat on circulating Aβ-40, LDL, and oxidized LDL in aged rats.** Aged rats were treated daily for 2 weeks with cinaciguat (0.3 mg/kg i.g.; *open squares*) or solvent control (inverted *triangles*), and results compared to levels in young rats (*closed circles*) as measured by ELISA. *A*, plasma Aβ-40; *B*: serum LDL; *C*: serum oxidized LDL. Figure 2*A*: One-way ANOVA was significant (*p* = 0.0018) with Tukey’s multiple comparison tests as follows: young *versus* aged rat LSEC CM *p* = 0.0021; aged rat ± cinaciguat LSEC CM *p* = 0.0047. Figure 2*B*: One-way ANOVA was significant (*p* < 0.0001) with Tukey’s multiple comparison tests as follows: young *versus* aged rat LSEC CM *p* = 0.0012; LSEC CM aged rat ± cinaciguat *p* = 0.0024. Figure 2*C*: One-way ANOVA was significant (*p* < 0.0001) with Tukey’s multiple comparison tests as follows: young *versus* aged rat LSEC CM *p* < 0.0001; Young rat *versus* cinaciguat-treated aged rat LSEC CM; *p* = 0.0065. LSEC CM aged rat ± cinaciguat *p* < 0.0001. n = 3 to 4 biological replicates. LSEC, liver sinusoidal endothelial cell.

Consistent with previous findings that serum LDL increases with age, we observed a two-fold increase in serum native LDL in aged rats compared to young rats (Fig. 2*B*); serum oxidized LDL increases 2.8 fold (Fig. 2*C*), reflecting longer LDL residence time in the circulation that offers opportunity for structural modification (27) and the higher oxidative stress associated with aging (28). Intriguingly, cinaciguat treatment decreases age-related elevations of both native and oxidized LDL (Fig. 2, *B* and *C*).

Cinaciguat's effect on aged rats' serum oxidized LDL levels may be explained in terms of its direct effect on LSECs themselves. As circulating LDL undergoes structural modifications such as glycation, oxidation, and desialylation 27, 28, 29, it becomes progressively more electronegative (30), which increases its binding affinity to LSECs’ Stabilin-1 and -2 receptors (31), the LSECs' workhorse scavenger receptors. Given our data that cinaciguat enhances aged rat LSEC endocytic capacity for FITC-labeled formaldehyde-treated serum albumin (Fig. 1*C*), cinaciguat's impact on aged rat serum oxidized LDL may be explained through recovery of LSEC endocytic capacity.

### Young, but not aged, LSECs enable hepatocyte Aβ and LDL uptake

Because existing literature demonstrated that LSEC defenestration does not increase circulating LDL levels (12) and LSECs do not take up LDL (31), we searched for alternative mechanisms to account for our observation that cinaciguat normalized aged rats’ circulating levels of native LDL. We hypothesized that cinaciguat acted through an indirect effect on nonparenchymal cells, since hepatocytes do not express sGC (32) and therefore were not direct targets for cinaciguat; within the nonparenchymal fraction, LSECs have been previously shown to maintain primary hepatocytes’ LDL uptake *in vitro* (33), offering a basis to investigate LSEC-hepatocyte crosstalk specifically.

In order to investigate whether pseudocapillarized LSECs lost the ability to promote hepatocyte LDL uptake, we isolated LSECs from young rats and from aged rats treated for 2 weeks with either solvent control or cinaciguat. We plated LSECs and placed cells on an orbital shaker to maintain their exposure to fluid shear stress and cultured overnight with VEGF, 20 ng/ml. The next day, we aspirated the LSEC-conditioned medium (LSEC-CM) and replaced the culture medium of freshly plated hepatocytes with LSEC-CM. All hepatocytes were isolated from young rats to avoid confounding effects of hepatocyte aging, and hepatocytes were sex-matched with the LSECs used to generate the conditioned medium.

Consistent with previous observations, hepatocytes in solo 2D monolayer culture did not take up the fluorescent-labeled LDL, DiI-LDL (Fig. 3, *A* and *B*) 33, 34. Hepatocytes in young rat LSEC-CM showed robust labeled DiI-LDL uptake over three hours, demonstrating that conditioned medium replicates the effects of co-culture as observed by Nahmias *et al.* (33). Importantly, we show for the first time that this paracrine effect is lost with pseudocapillarization as hepatocytes failed to take up DiI-LDL when cultured with LSEC-CM from pseudocapillarized LSECs (Fig. 3, *A* and *B*). Moreover, LSECs from aged rats treated with cinaciguat regain normal function, as their conditioned medium rescued hepatocyte DiI-LDL uptake. Indeed, our endocytic uptake studies demonstrate that hepatocytes in pseudocapillarized LSEC-CM take up no more DiI-LDL than hepatocytes in control medium, while there is no significant difference between hepatocytes in young LSEC-CM and in LSEC-CM from cinaciguat-treated aged rats (Fig. 3*B*).

**Figure 3:**
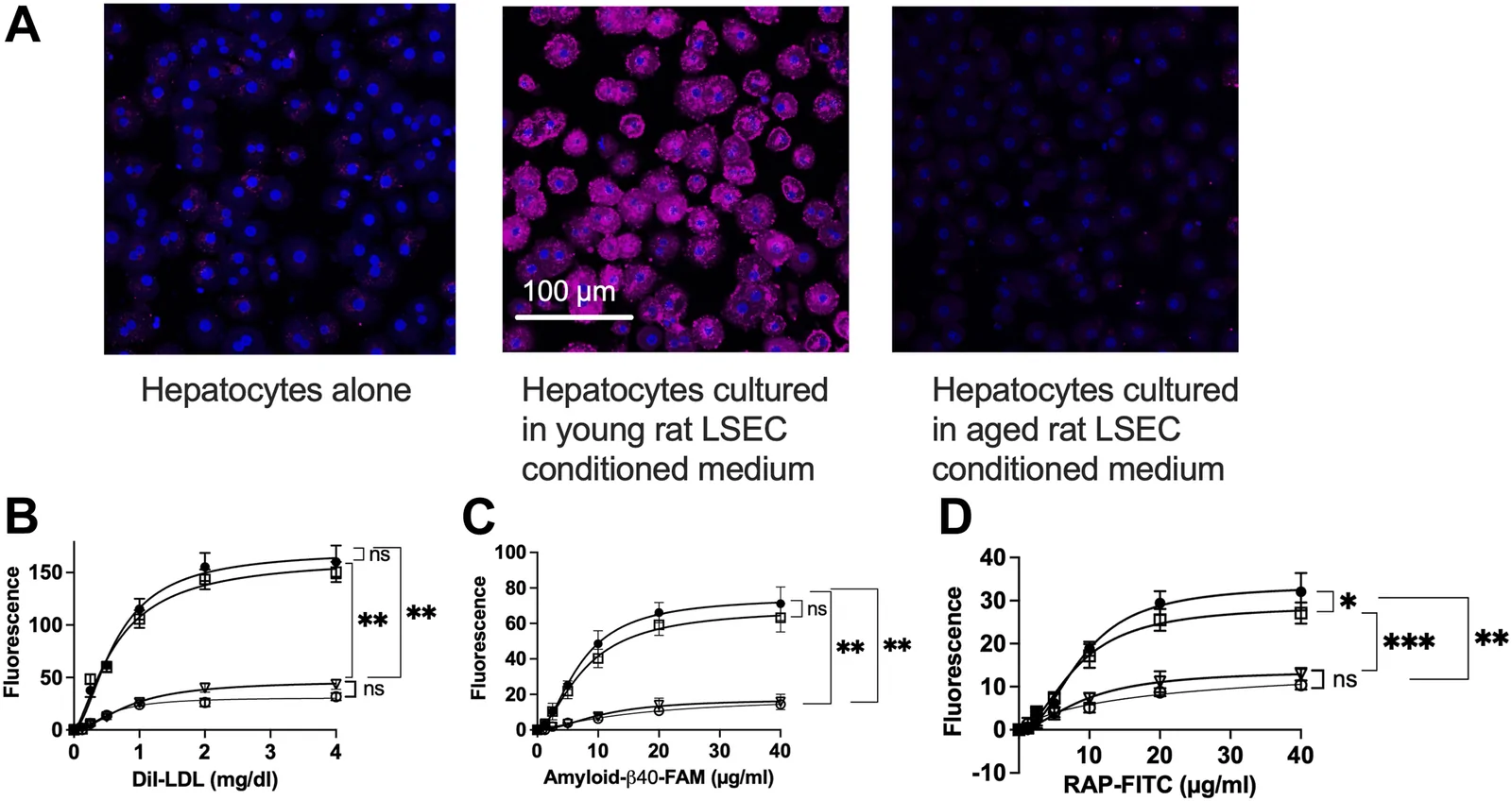
**Hepatocyte endocytosis *ex vivo* in the presence or absence of LSEC-CM.***A*, hepatocytes cultured for three hours in the presence of DiI-LDL (*magenta*) in control DMEM (*left panel*), in young rat LSEC-CM (*center*), and in aged rat LSEC-CM (*right panel*). Nuclear stain with DAPI (*blue*). Hepatocyte endocytic capacity for: (*B*) DiI-LDL, (*C*) amyloid-β40-FAM labeled, (*D*) receptor-associated protein-FITC. Young rat hepatocytes cultured for three hours in control DMEM (*open circles*), young rat LSEC-CM (*closed circles*), aged rat LSEC-CM (*inverted open triangles*), or aged cinaciguat-treated rat LSEC-CM (*open squares*). Figure 3*B*: Endocytic capacity was plotted in saturation-binding plots that were found to best fit either a Michaelis-Menten or an allosteric sigmoidal model; Vmax (maximal internalization rate) was compared between groups: Young rat LSEC CM *versus* aged rat LSEC CM *p* = 0.0013; LSEC CM aged rat ± cinaciguat *p* = 0.0017. Figure 3*C*: Endocytic capacity was plotted in saturation-binding plots that were found to best fit either a Michaelis-Menten or an allosteric sigmoidal model; Vmax (maximal internalization rate) was compared between groups. Young rat LSEC CM versus aged rat LSEC CM *p* = 0.0325; LSEC CM aged rat ± cinaciguat *p* = 0.018.: Figure 3*D*: Endocytic capacity was plotted in saturation-binding plots that were found to best fit either a Michaelis-Menten or an allosteric sigmoidal model; Vmax (maximal internalization rate) was compared between groups. Young rat LSEC CM *versus* aged rat LSEC CM *p* = 0.0010; LSEC CM aged rat ± cinaciguat *p* = 0.0004. n = 6 biological replicates. No significant differences were found between the sexes by one-way ANOVA. LSEC-CM, LSEC-conditioned medium.

Interestingly, these phenomena extend beyond LDLR-mediated uptake of DiI-LDL. Hepatocytes cultured in young LSEC-CM or in LSEC-CM from cinaciguat-treated aged rats showed significantly enhanced uptake of LRP1 ligands compared to hepatocytes cultured in control medium or pseudocapillarized LSEC-CM (Fig. 3, *C* and *D*). These ligands include the classic LRP1 receptor-associated protein (RAP) and recombinant Aβ40 itself, which can be taken up by LRP1 either as part of a chylomicron remnant (as it is *in vivo*) or on its own through its 17-24th residues, which act as a recognition sequence for LRP1-mediated uptake (35). Neutralizing antibody to LRP1 completely blocked the paracrine effect of young LSEC-CM on hepatocyte uptake of Aβ (Fig. 4). Collectively, these data suggest that cinaciguat's ability to reduce circulating plasma Aβ40 may not only derive from improved chylomicron remnant access to the sinusoidal space, but also by restoring paracrine signaling that increases hepatocyte endocytic capacity.

**Figure 4:**
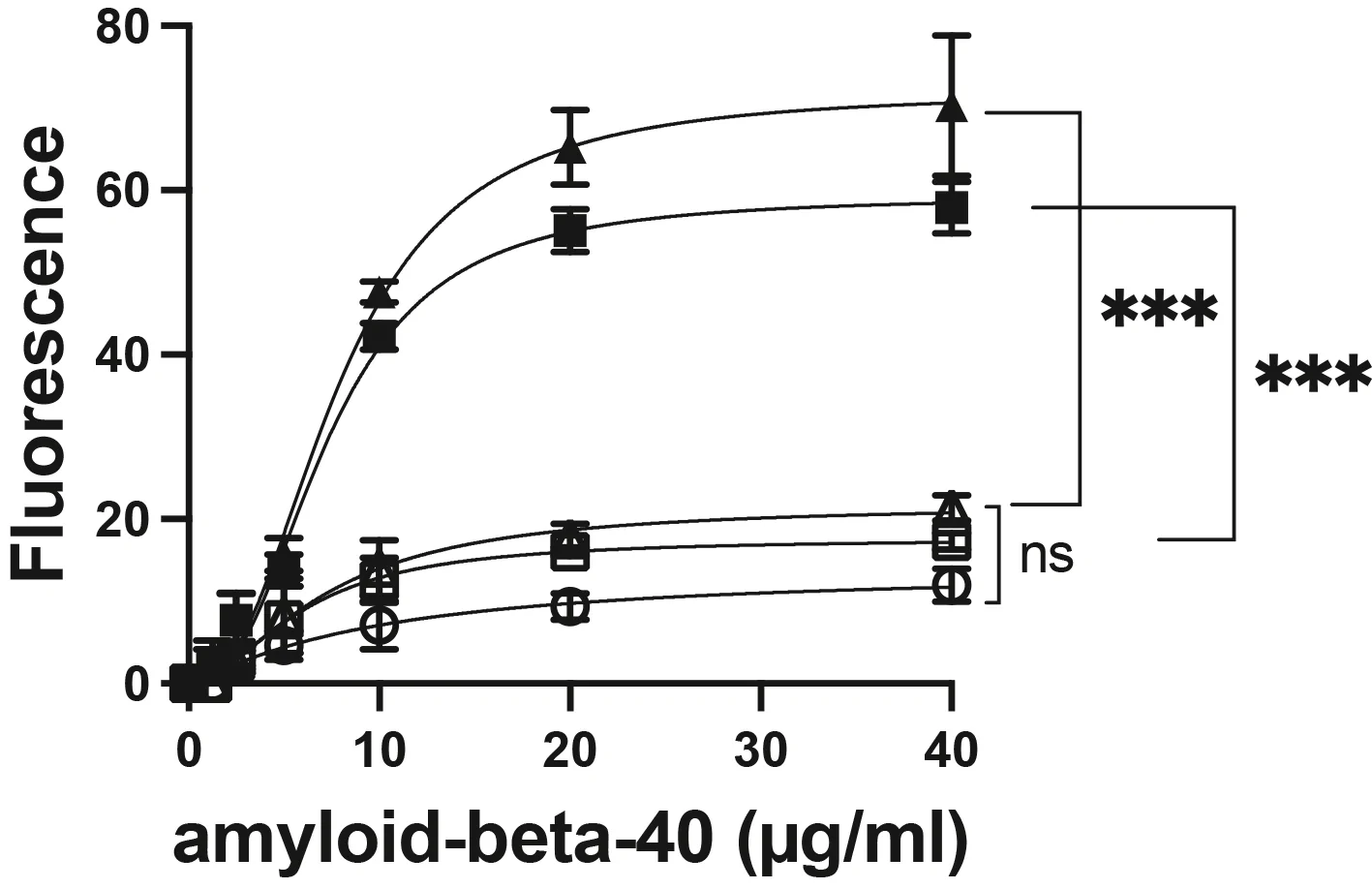
**Effect of neutralizing antibody to LRP1 on the paracrine effect on hepatocyte uptake of amyloid-beta.** Quantified fluorescence of Aβ-40-FAM taken up over three hours by hepatocytes from young rats cultured in LSEC-CM with (*open triangles*) or without (*closed triangles*) anti-LRP1, or in DMEM spiked with 50 ng/ml recombinant HB-EGF with (*open square*) or without (*closed square*) anti-LRP1, or DMEM alone (*open circle*). Endocytic capacity was plotted in saturation-binding plots that were found to best fit either a Michaelis-Menten or an allosteric sigmoidal model; Vmax (maximal internalization rate) was compared between groups. Young rat LSEC CM ± anti-LRP1 *p* < 0.0001; HB-EGF ± anti-LRP1 *p* < 0.0001. n = 3 biological replicates. HB-EGF, heparin-binding epidermal growth factor-like growth factor; LSEC-CM, LSEC-conditioned medium.

### HB-EGF mediates age-associated change in LSEC-hepatocyte signaling

We next sought to identify the molecular mediator of the paracrine signaling. Because elements of age-related LSEC pseudocapillarization phenocopy disease-induced LSEC capillarization, we investigated whether pseudocapillarized LSECs, like capillarized LSECs (20), were unable to secrete heparin-binding epidermal growth factor-like growth factor (HB-EGF) and, if so, whether this accounted for their inability to maintain hepatocyte LDLR and LRP1 ligand uptake.

We therefore isolated LSECs from young rats and from aged rats treated with 2 weeks of either cinaciguat or solvent control. We incubated these cells overnight, and then measured soluble HB-EGF (sHB-EGF) in the supernatant by ELISA (Fig. 5*A*). Much like capillarized LSECs in liver disease, pseudocapillarized LSECs did not secrete detectable sHB-EGF, in contrast to sHB-EGF secretion by young healthy LSECs. Pseudocapillarized LSECs' impaired sHB-EGF release was not purely a failure of synthesis: immunoblots demonstrated pro-HB-EGF protein expression in pseudocapillarized LSECs, whereas young, healthy LSECs retained minimal pro-HB-EGF (Figs. 5, *B* and *C*, supporting S3). Together, these results suggest that young LSECs synthesize and rapidly release sHB-EGF (accounting for the low cellular pro-HB-EGF expression but detectable levels in supernatant). In contrast, pseudocapillarized LSECs retain rather than secrete sHB-EGF. These dynamics in isolated, pseudocapillarized LSECs mirror capillarized LSECs' HB-EGF disposition in chronic liver disease (20) and is recapitulated *in vivo*: confocal immunofluorescence demonstrates that LSECs in young liver do not retain pro-HB-EGF, but in sections from aged liver, LSECs do retain pro-HB-EGF (Fig. 5, *D* and *E*).

**Figure 5:**
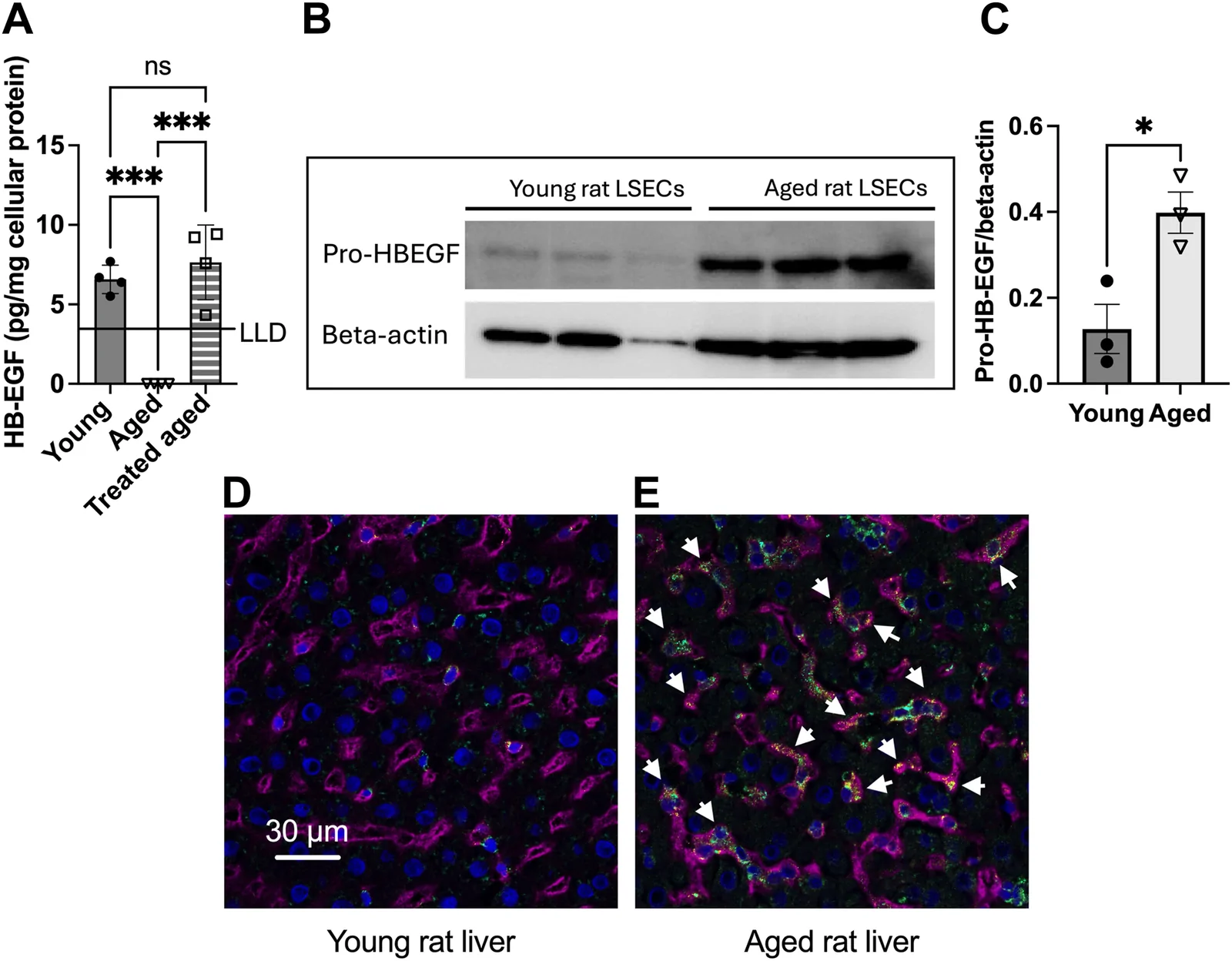
**Changes in HB-EGF secretion and disposition with aging.***A*, HB-EGF in cell culture supernatant from overnight culture of LSECs from young (*closed circles*), aged (*inverted open triangles*), and aged cinaciguat-treated rats (*open squares*). Lower limit of detection 2.26 pg/mg cellular protein. n = 4. *B*, immunoblot for pro-HB-EGF in freshly isolated LSECs from young rats and aged rats. *C*, quantitation of pro-HB-EGF with beta-actin control in *panel B*. *D and E*, confocal microscopy of liver sections from young (*D*) and aged (*E*) rats. HB-EGF (*cyan*), endothelial marker CD31 (*magenta*), and DAPI (*blue*). *Arrows* indicate *yellow* costaining of HB-EGF in LSECs. Figure 5*A*: One-way ANOVA was significant(*p* < 0.0001) with Tukey’s multiple comparisons test as follows: young rat LSEC CM *versus* aged rat LSEC CM *p* = 0.0003; aged rat LSEC ± cinaciguat *p* = 0.0001. Figure 5*C*: Pro-HBEGF/beta actin young rat LSECs *versus* aged rat LSEC *p* = 0.022 by unpaired *t* test. n = 3 biological replicates. LSEC-CM, LSEC-conditioned medium; HB-EGF, heparin-binding epidermal growth factor-like growth factor.

Treating aged rats with cinaciguat restored aged LSECs' sHB-EGF release into cell culture supernatant (Fig. 5*A*). Moreover, the resumption of sHB-EGF release upon sGC activation treatment mirrors the response of other signs of pseudocapillarization (Fig. 1).

Because the dynamics of LSEC sHB-EGF release fit the pattern observed in Figures 2 and 3, we tested it as a candidate mediator of LSEC-drive hepatocyte LDLR and LRP1-dependent ligand uptake (Fig. 6). Supplementing the culture medium of primary hepatocytes from young rats with recombinant HB-EGF replicated the effects of culture in young LSEC-CM in terms of promoting endocytic uptake of DiI-LDL, Aβ - fluorescein amidites (FAM), and RAP-FITC (Fig. 6, *A*–*C*). Conversely, anti-HB-EGF neutralizing antibody abrogated the effects of LSEC-CM from young rats on hepatocyte uptake of DiI-LDL, Aβ-FAM, and RAP-FITC (Fig. 5, *D*–*F*). Together, these experiments suggest that HB-EGF is the principal, if not exclusive, mediator of this novel axis of LSEC-hepatocyte signaling that is lost upon LSEC pseudocapillarization, but restored when pseudocapillarization is reversed with sGC activation.

**Figure 6:**
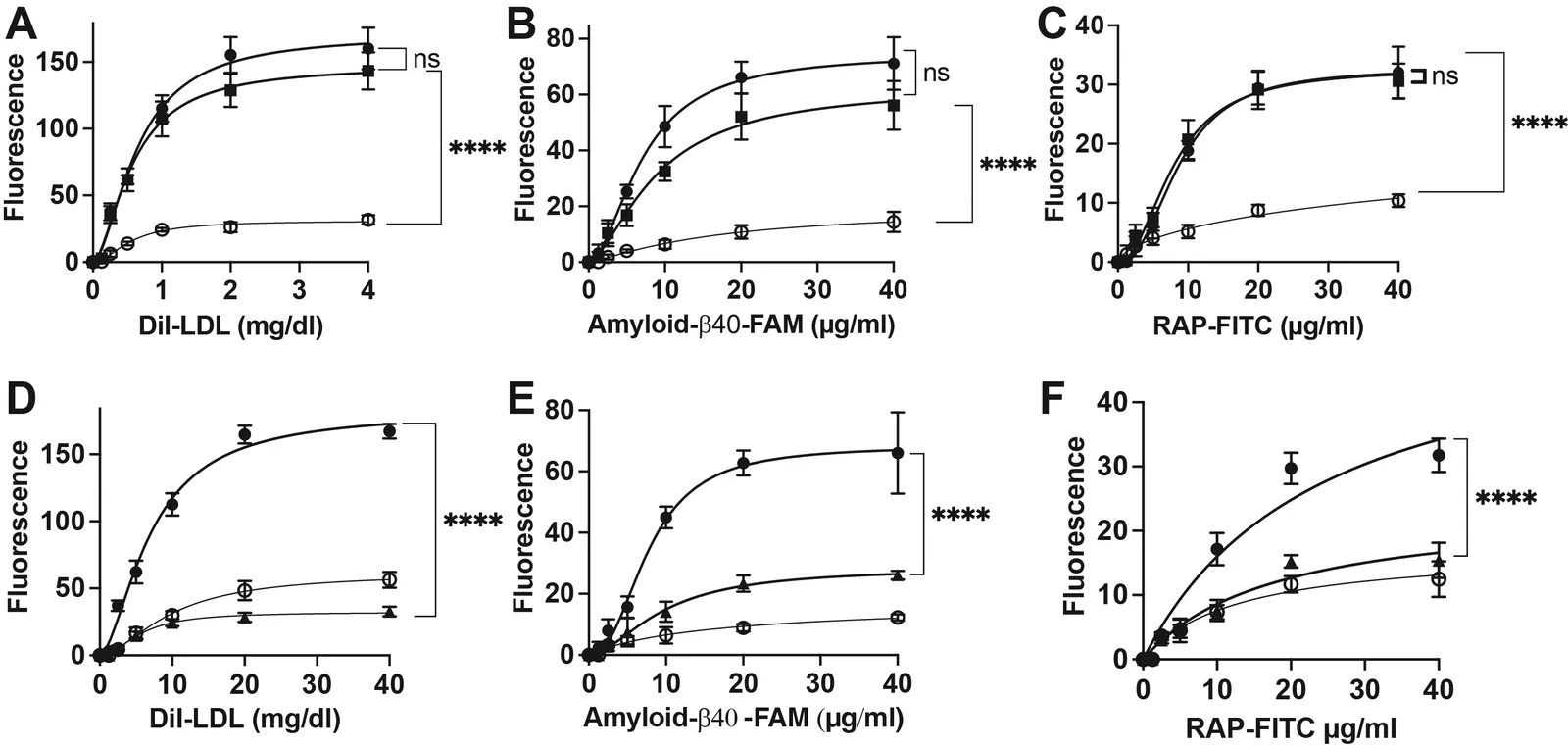
**Effect of HB-EGF on hepatocyte endocytic capacity *ex vivo*.***A*–*C*, quantified fluorescence of ligand taken up over three hours by hepatocytes from young rats cultured in control DMEM (*open circles*), young rat LSEC-CM (*closed circles*), or DMEM spiked with 50 ng/ml recombinant HB-EGF (*closed squares*). *D*–*F*, quantified fluorescence of ligand taken up over three hours by hepatocytes from young rats cultured for three hours in control DMEM (*open circles*), young LSEC-CM spiked with (*closed triangles*) or without (*open circles*) 10 ng/ml HB-EGF neutralizing antibody. *A*–*C*, n = 6 biological replicates, no differences were found between the sexes by one-way ANOVA. *D*–*F*, n = 3 biological replicates. For Figure 6, *A*–*C*, endocytic capacity was plotted in saturation-binding plots that were found to best fit either a Michaelis-Menten or an allosteric sigmoidal model; Vmax (maximal internalization rate) was compared between groups. DMEM ± recombinant HB-EGF *p* < 0.0001; for Figure 6, *D–F* endocytic capacity was plotted in saturation-binding plots that were found to best fit either a Michaelis-Menten or an allosteric sigmoidal model; Vmax (maximal internalization rate) was compared between groups: young rat LSEC CM ± HB-EGF neutralizing antibody *p* < 0.0001. LSEC-CM, LSEC-conditioned medium; HB-EGF, heparin-binding epidermal growth factor-like growth factor.

### Cinaciguat treatment restores hepatocyte LDLR and LRP1 expression at the plasma membrane

To connect *in vivo* phenomena to our *ex vivo* findings about pseudocapillarized LSECs and hepatocyte LDLR and LRP1-mediated endocytosis, we isolated hepatocytes from young rats and aged rats treated with either cinaciguat or solvent control. In addition, we further isolated hepatocyte plasma membrane specifically. Protein expression assays revealed that whole cell protein expression of hepatocyte LDLR and LRP1 remain stable with aging (Figs 7*A*, supporting S4). By contrast, hepatocytes from aged control rats have significantly lower LDLR and LRP1 expression on the plasma membrane compared to hepatocytes from young rats or from cinaciguat-treated aged rats (Figs 7*B*, supporting S4). Strikingly, our experiments find that aging does not alter LDLR or LRP1 protein abundance, but rather changes protein localization. The defect lies not in protein expression, but in subcellular localization. Because hepatocytes express little to no sGC themselves (32), cinaciguat cannot act on them directly. Instead, its restorative effect on LDLR and LRP1 localization is through an indirect action on nonparenchymal cells that ultimately reprograms hepatocytes’ LDLR and LRP1 receptor localization.

**Figure 7:**
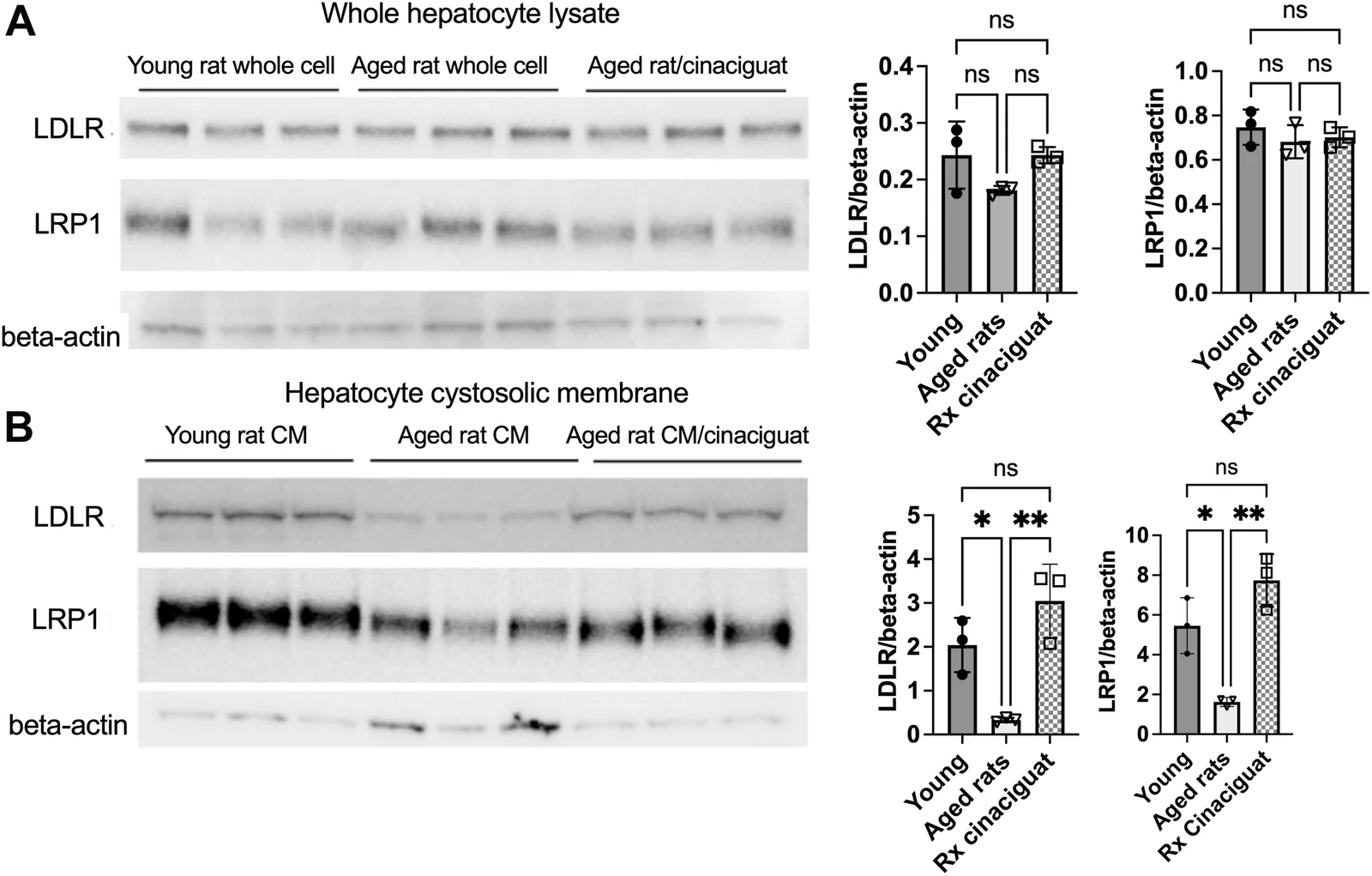
**Change in hepatocyte whole cell *versus* surface LDLR and LRP1 expression with aging with and without sGC activator treatment.***A*, immunoblot for LDLR and LRP1 in hepatocyte whole cell lysates. Quantitation of LDLR (*center*) and LRP1 (*right panel*) with beta-actin control in hepatocytes from young rats (*closed circles*, *dark column*), aged rats (*inverted triangles*, *lighter gray column*), and aged cinaciguat-treated rats (*open squares*, *hatched column*). *B*, immunoblot for LDLR and LRP1 in hepatocyte cytoplasmic membrane isolated from young, aged control, and aged cinaciguat-treated rats. LDLR (*center*) and LRP1 (*right panel*) normalized with beta-actin control (*right panel*). Figure 7*A*: comparisons were not significant by one-way ANOVA. Figure 7*B*: One-way ANOVA was significant for LDLR (*p* = 0.0042) and for LRP1 (*p* = 0.0016) with Tukeys multiple comparisons test as follows: surface expression LDLR young *versus* aged rat hepatocytes=0.0307, surface expression aged LDLR rat hepatocytes ± cinaciguat *p* = 0.0036; surface expression LRP1 young *versus* aged rat hepatocytes=0.0138, surface expression LRP1 aged rat hepatocytes ± cinaciguat *p* = 0.0013. n = 3 biological replicates. LRP1, low-density lipoprotein receptor-related protein-1.

LRP1 is the main receptor involved in brain Aβ clearance across the BBB and is thought to play a major role in hepatic clearance (36). However, LDLR is the major receptor responsible for Aβ uptake by astrocytes (37). To confirm that the LSEC paracrine effect on hepatocyte clearance was not limited to LDLR, but also acted on hepatocyte LRP1, we examined the effect of a neutralizing antibody to LRP1 on hepatocyte uptake of Aβ in the presence of young rat LSEC-CM or recombinant HB-EGF (Fig. 4). Stimulation of hepatocyte Aβ uptake by either LSEC-CM or HB-EGF was completely abrogated in the presence of anti-LRP1, indicating that the paracrine effect of LSEC HB-EGF on hepatocyte Aβ uptake was indeed through stimulation of the LRP1 pathway.

Though these results cannot directly link LSEC HB-EGF to alterations in hepatocyte LDLR or LRP1 localization, they strongly support an HB-EGF-mediated effect. Likewise, rapid enhancement of hepatocyte endocytic uptake over the course of just three hours of incubation with HB-EGF/LSEC-CM is entirely consistent with HB-EGF-induced LDLR and LRP1 translocation to the plasma membrane and/or more efficient endocytic receptor recycling that results in greater accumulation of receptors at the cell surface.

## Discussion

These studies establish that activating sGC *in vivo* is sufficient to reverse LSEC pseudocapillarization. Just 2 weeks of cinaciguat treatment is sufficient to restore LSEC fenestration, improve LSEC endocytic capacity, and normalize release of sHB-EGF. Furthermore, these studies show that HB-EGF itself is a previously undescribed paracrine promoter of hepatocyte LDL and Aβ uptake and mediates the previously-observed benefits of LSEC-hepatocyte coculture with respect to LDL uptake (33). Indeed, co-culture with LSECs has been shown to prolong the expression of hepatocyte phenotypic features 38, 39 and modulate the hepatocyte phenotype (40). Our findings are the first to establish a direct link between age-related alterations in hepatic lipoprotein receptor localization and regulation by the sGC-cGMP pathway. This previously unrecognized pathway opens an entirely new framework for understanding hepatic lipid handling in aging and warrants rigorous investigation into the underlying molecular mechanisms: specifically, the role of HB-EGF/EGFR signaling in governing receptor dynamics *in vivo*.

The present work demonstrates that treatment with cinaciguat results in a substantial decrease in circulating levels of Aβ, LDL, and oxidized LDL in a model of healthy aging to levels comparable to those seen in young rats. Because sGC activators specifically target dysfunctional oxidized/heme-free sGC, they are expected to be more effective than sGC stimulators in a disease milieu with high oxidative stress (*e.g.* aging, diabetes, metabolic-dysfunction associated fibrotic liver disease (41)). While the effects of cinaciguat were dramatic even on a relatively short, 2-week time scale, future studies should explore whether these reductions in healthy aging translate to meaningful improvements in a disease model. Given the striking effects demonstrated in our study, not only on Aβ, but also on atherogenic LDL and oxidized LDL 42, 43, 44, 45, 46, sGC activators merit study both in models of Alzheimer's disease and vascular dementia, a disease area with promising preclinical data for sGC agonism (47).

LSECs express phosphodiesterases, such as PDE5, that act on the NO-sGC-cGMP pathway to degrade cGMP, and LSECs are responsive to PDE5 inhibition (48); whereas sGC agonists increase intracellular cGMP by increasing cGMP synthesis, PDE5 inhibitors increase intracellular cGMP by decreasing cGMP degradation. By implication, findings from the present studies, which use an sGC activator, may elucidate a previously-undescribed peripheral mechanism involved in PDE5 inhibitors' protection against cognitive impairment and Alzheimer pathology, as observed in a large body of existing literature 49, 50, 51, 52. Future studies may investigate whether sGC agonism or phosphodiesterase inhibition is a more efficient means of increasing intracellular cGMP in humans.

One consideration in investigating the translational relevance of these findings will be to utilize appropriate disease models based on the putative mechanism identified in this work: namely, reversing pseudocapillarization and restoring LSEC-hepatocyte crosstalk. Traditional transgenic animal models of Alzheimer's and atherosclerotic cardiovascular disease (ASCVD) conventionally rely on young animals, which necessarily lack age-related pseudocapillarization. Though transgenic mouse models have long been favored for their overt neurodegenerative phenotype and high rates of Aβ synthesis, when assessing the hepatic contributions to AD progression, transgenic models have been linked to confounding artifacts that range from disruptive to ruinous 36, 53. APP/PS1 mutations reduce hepatic LDLR and LRP1 by as much as 80 to 90% 36, 53—the very receptors responsible for peripheral Aβ clearance—which could nullify therapeutic approaches that rely on enhancing hepatic clearance.

Though no commonly used *in vivo* models faithfully recapitulate both age-related LSEC impairment and ASCVD/Alzheimer's pathology, the translational relevance may justify the challenge. LSEC pseudocapillarization is an attractive and clinically relevant therapeutic target for both ASCVD and Alzheimer's. Both diseases are highly prevalent in older adults 54, 55, 56, in whom pseudocapillarization is near-universal after age 60 (Supporting Fig. S1) (14). Moreover, targeting LSEC pseudocapillarization may yield particular benefits in Alzheimer's disease: the liver is vastly more accessible to pharmacologic intervention, whereas the blood-brain barrier has hindered decades of drug development. Alzheimer's therapies have had a 98% clinical trial failure rate since 2004, substantially higher than other disease areas 57, 58, and a strategy aimed at peripheral therapeutic targets may have more success.

Recent studies have demonstrated that hepatic fibrosis and metabolic dysfunction-associated liver disease are risk factors for cognitive decline and dementia 59, 60, 61, 62. LSEC capillarization is permissive for and precedes hepatic fibrosis, including in metabolic dysfunction-associated liver disease 63, 64, 65. Many of the features of LSEC pseudocapillarization in aging are phenocopied in disease-induced LSEC capillarization, including reduced LSEC permeability and sHB-EGF secretion 13, 20, and the same treatment used in this study to reverse pseudocapillarization has also been shown to reverse LSEC capillarization (18). These similarities raise the possibility that dementia risk associated with chronic liver disease may be mitigated by restoring LSECs to normal phenotype.

In conclusion, our work identified a previously undescribed LSEC-hepatocyte signaling axis in which LSEC HB-EGF serves as a paracrine regulator of hepatocyte uptake of Aβ and LDL uptake in cells from young liver. We show that aging disrupts this pathway through pseudocapillarization and impaired HB-EGF secretion. We further demonstrate, for the first time, that short term administration of sGC activator reverses LSEC pseudocapillarization, normalizes circulating Aβ and LDL, restores LSEC HB-EGF secretion, and reestablishes hepatocyte LDLR and LRP1 plasma membrane expression in aged rats, allowing for LSEC paracrine regulation of hepatocyte uptake of Aβ and LDL. Collectively, these findings define a new, therapeutically actionable mechanism linking hepatic vascular aging to dysregulated lipid and Aβ homeostasis, with broad implications for cardiovascular and neurodegenerative disease.

## Experimental procedures

### Antibodies and reagents

All antibodies used were commercially validated. Information on antibodies can be found in supporting Table S1. Commercial reagents and kits are listed in supporting Table S2.

### Animal studies

All animal experiments were conducted according to protocols approved by the Keck School of Medicine Animal Care and Use Committee, protocol number 21453. All the animals received humane care in accordance as per the Guide for the Care and Use of Laboratory Animals prepared by the National Academy of Sciences. All studies performed with n = 6 included 3 male and 3 female rats.

4- and 24-month-old male and female Fischer rats were obtained from Charles Rivers laboratories and from by the NIAAA aging rodent colony, respectively.

### Cell isolation

Detailed protocols of cell isolation are provided in the supporting methods. LSECs were isolated by density gradient centrifugation and elutriation 19, 66. Hepatocytes were isolated by collagenase perfusion and purified by Percoll density centrifugation 19, 66.

### Hepatocyte uptake studies

Hepatocytes were plated on coverslips coated with rat tail collagen. Hepatocytes were cultured in the presence of Di-I-labeled LDL, FAM labeled amyloid-beta (1-40), FITC-labeled LRP1 receptor-associated protein (RAP), recombinant HB-EGF, 50 ng/ml, or anti-HB-EGF neutralizing antibody, 10 ng/ml, for 3 h and then fixed with 4% paraformaldehyde. Coverslips were attached to slides with a mounting medium containing DAPI. Fluorescence images were taken of 10 to 15 fields by Leica TCS SP8 confocal microscope and analyzed with ImageJ software (https://imagej.net/ij/download.html) that calculated the area of fluorescent stain.

### Endocytic capacity

#### Conditioned medium

LSECs from 4- or 24-month-old rats were plated in DME-low glucose with 10% fetal bovine serum. After 2 h the culture medium was changed to DMEM-high glucose and LSECs were cultured overnight on a rotary shaker with a calculated shear stress of 1.26 dyn/cm^2^. Conditioned medium was collected after 16 h.

#### Incubations

Hepatocytes isolated from 4-month-old rats were plated in DMEM with 10% fetal bovine serum in 24-well plates coated with rat tail collagen. Two hours after plating the hepatocytes, the medium was changed to DMEM or to medium conditioned by rat LSECs matched for sex.

Hepatocytes were cultured in the presence of 1,1′-Dioctadecyl-3,3,3′,3′-Tetramethylindocarbocyanine Perchlorate (Di-I)-LDL, amyloid-beta (1-40)- FAM, RAP-FITC, recombinant HB-EGF, 50 ng/ml, or anti-HB-EGF neutralizing antibody, 10 ng/ml, for 3 h. Fluorescence images were taken by Leica TCS SP8 confocal microscope and analyzed with ImageJ.

### cGMP measurement

We used the Cyclic GMP ELISA Kit (Cayman Chemical cat#581021) according to manufacturer’s instructions and final cGMP quantification was normalized to total protein.

### Immunoblotting

Supernatant protein concentration was measured using a Bradford protein spectrophotometric assay kit (Bio-Rad). Immunoblots for hepatocyte plasma membrane were performed on lysates of plasma membrane isolated with the Pierce Cell Surface Protein Isolation Kit. Antibodies and reagents are listed in supporting Tables S1 and S2. Membranes were incubated with the desired primary antibodies overnight followed by secondary antibody for 1 h. Proteins were visualized using Luminol enhanced chemiluminescent reagent on a ChemiDoc MP Imaging System (Bio-Rad). Image analysis, densitometry, and gray level calculation for each band in pixels were normalized to a reference band (depicted under immunoblot images) using Image Lab Software with background subtracted per the Bio-Rad manual. Scans of full-length original western blots are available in the supporting material.

### Scanning electron microscopy

LSECs plated on collagen-coated Thermanox coverslips were fixed with 2% glutaraldehyde in 0.1 M cacodylate buffer, pH 7.4 for 30 min. After washing, the slides were treated with 1% tannic acid (Electron Microscopy Sciences) in 0.15 M cacodylate buffer for 1 h, postfixed with 1% osmium tetroxide (Ted Pella) in 0.1 M cacodylate buffer for 30 min, dehydrated with graded alcohols, dried with hexamethyldisilazane (Ted Pella), sputtercoated with 10-nm gold, and examined *via* a JSM-6390LV scanning electron microscope (JEOL). Porosity, the percentage of the cell surface covered by fenestrae, was quantified by ImageJ: total LSEC surface area with exclusion of the nucleus and the open fenestrated area with exclusion of gaps were measured and the porosity was calculated as percent of open area.

### Statistics

Data were analyzed and presented as mean ± sd. GraphPad Prism v.11.0.1 was used for statistical analyses and graphing. Endocytic capacity was plotted in saturation-binding plots that were found to best fit either a Michaelis-Menten or an allosteric sigmoidal model; Vmax (maximal internalization rate) was compared between groups. One-way ANOVA was significant for all studies where *p*-values are shown for between *a posteriori* comparisons, except for immunoblot quantification, which was analyzed with unpaired *t* test. *p* < 0.05 was considered statistically significant. All studies were performed with n = 3 to 6, with 3 male and 3 female rats for studies with n = 6. All individual data points are provided in Table S3.

## Data availability

All data are presented within the manuscript or in supporting information.

## Supporting information

This article contains supporting information (14, 19, 66).

## Conflict of interest

The authors declare that they have no conflicts of interest with the contents of this article.
