# Supplementary material for: Reversing liver sinusoidal endothelial aging restores clearance of amyloid-beta and LDL
Source: J Biol Chem. 2026 Sep 26;302(11):113608. doi: 10.1016/j.jbc.2026.113608 (PMC13720804; doi:10.1016/j.jbc.2026.113608)
Supplement: Supplementary file 1 [file mmc1.docx]

**Hepatocyte clearance of amyloid-beta and LDL requires liver sinusoidal endothelial cell fenestration and paracrine signaling**

Sam J. de Leve, Xiangdong Wang, Helia Alavifard, Lily Dara, Laurie D. DeLeve

**Table of contents supporting material**

Supporting material: Experimental procedures and materials

Supporting Table S1. List of antibodies

Supporting Table S2. Commercial reagents and assay kits.

Supporting Table S3. Individual data points for all figures

Supporting Fig S1. Pseudocapillarization in humans.

Supporting Fig S2. Scanning electron microscopy of fenestration of LSEC from young rats and

from aged rats treated with solvent control or with cinaciguat.

Supporting Fig S3. Full unedited blot for Fig 4.

Supporting Fig S4. Full unedited blot for Fig 6.

**Supporting material: Experimental procedures and materials**

**Detailed protocol for isolation of rat liver sinusoidal endothelial cells.**

LSECs were isolated by density gradient centrifugation and elutriation as previously described ^[1, 2]^; . Rats were sedated with ketamine/xylazine and then treated with 200 µl porcine intestine heparin (1,000 units/ml). The liver was perfused for 10 min with calcium-free Gey’s buffered saline (GBS) at room temperature at 10 ml/min, followed by perfusion in a recirculating fashion for 20 min with GBS containing 0.05% type 1a collagenase. The livers were mechanically dissociated, pressed through polypropylene mesh, and centrifuged, and the digest was resuspended in 50 ml GBS. The initial separation was achieved with a 17% iodixanol density gradient centrifugation. Elutriation was performed in a J2-21 Beckman centrifuge using the JE-6B rotor with standard chambers. The first elutriation was at 2,500 rpm and a pump speed of 27.8 ml/min. The first 100 ml of eluate was collected and centrifuged. The pellet was subjected to a second elutriation at 3,400 rpm. The first 100 ml were collected at a flow rate of 32 ml/min and discarded. Flow rate was increased to 61 ml/min, and 100 ml of eluate was collected and centrifuged. The average yield of LSECs was 85 million/rat with 95% purity and 97% viability.

**Detailed protocol for isolation of rat hepatocytes**

Hepatocytes were isolated by collagenase perfusion and purified by Percoll density centrifugation. Rats were anesthetized with ketamine/xylazine, the abdomen was opened, and 200 µl porcine intestine heparin (1,000 units/ml) was injected into the inferior vena cava. The chest was opened and a 16g cannula was inserted through the right atrium into the inferior vena cava. The portal vein was cut, the inferior vena cava below the liver was ligated, and oxygenated calcium-free Hank’s buffer was infused at 10 ml/min. After 10 minutes the buffer was changed to oxygenated Hank’s buffer with 0.035% type IV collagenase until the liver softened. The liver was mechanically dissociated, filtered through gauze mesh with 4°C Krebs buffer and allowed to settle by gravity. To enhance purity and viability, the pellet was subjected to Percoll density centrifugation as previously described. The average yield of hepatocytes was 250 million/rat with 89% viability.

| **Supporting Table S1. List of antibodies** | | | |
| --- | --- | --- | --- |
| Antibodies | Company | Cat. Number | Dilution or concentration |
| Beta-actin monoclonal antibody (AC-15) | Invitrogen | AM4302 | 1:1,000 |
| CD31 | R & D | AF3628 | 1:100 |
| LDLR | ThermoFisher | MA5-51226 | 1:1,000 |
| LRP1 | ThermoFisher | MA5-35053 | 1:5,000 |
| HB-EGF (immunoblot) | ThermoFisher | MA5-35148 | 1:1,000 |
| HB-EGF (staining) | Abcam | AB218019 | 1:100 |
| HB-EGF neutralizing antibody | R & D | AF-259 | 10 ng/ml |
| HRP Goat Anti-rabbit (H + L) secondary | Cell Signaling Technology | 7074 | 1:10,000 |

| **Supporting Table S2. Commercial reagents and assay kits used** | |
| --- | --- |
| **Reagents** | **Commercial vendor** |
| amyloid-beta (1-40)-FAM | Anaspec cat # AS-23514-01 |
| BAY 58-2667 (cinaciguat) | R&D Systems cat#6052 |
| DAPI | Invitrogen cat#D1306 |
| Di-I-LDL | Invitrogen cat#L3482 |
| FITC-FSA | D'Liver, Tromso Norway (no cat #) |
| Recombinant HB-EGF | R&D systems cat#259-HE-250. |
| glutaraldehyde | Electron Microscopy Sciences cat# [16535-05](https://www.emsdiasum.com/glutaraldehyde-0625-in-phosphate-buffer-ph-74) |
| hexamethyldisilazane | Ted Pella **cat# No.** 18605 |
| Iodixanol | Accurate Chemical and Scientific cat # **AN1114542** |
| Luminol enhanced chemiluminescent (ECL) reagent | ThermoFisher Scientific cat# WP20005 |
| osmium tetroxide | Ted Pella cat#18459 |
| Percoll | Sigma-Aldrich cat# P1644 |
| RAP-FITC | Abcam cat# Ab184738 |
| SlowFade Diamond | Invitrogen cat# S36967 |
| Type 1a collagenase | Sigma-Aldrich cat#C9891 |
| Type IV collagenase | Sigma-Aldrich cat# C4-28 |
| Recombinant mouse VEGF | R&D Systems cat no. 493-MV |
| **Commercial assay kits** | **Commercial vendor** |
| Alexa Fluor 488 conjugation kit | Abcam cat# ab236553 |
| Cyclic guanosine monophosphate (cyclic GMP) EIA Kit | Cayman cat#581021 |
| Pierce™ Cell Surface Protein Isolation Kit | ThermoFisher cat#89881 |
| Rat Amyloid Beta Peptide 1-40(Ab1-40) Elisa kit | MyBiosource cat#MBS2022403 |
| Rat HB-EGF ELISA kit | MyBioSource cat# MBS722484 |
| Rat low density lipoprotein (LDL) Elisa Kit | MyBiosource Cat# MBS 702165 |

^[3]^

**Supporting Fig S2**

**Scanning electron microscopy of fenestration of LSECs from young rats, aged rats, and cinaciguat-treated rats.** Enlargement of black outlined areas are shown to the right of their respective pictures. **Top panel**: scanning electron microscopy of two LSECs from a 2-month old F344 rat. Note fenestrae are grouped in sieve plates. Gaps seen in the cytoplasm are fixation artefacts. Nuclei are visible in the top right and left corners. **Middle panel**: scanning electron microscopy of LSEC from 24-month-old F344 rat. There are no visible fenestrae. **Bottom panel:** scanning electron microscopy of LSEC from a 24-month-old F344 rat treated with two weeks of cinaciguat, 0.3 mg/kg intragastric. Numerous fenestrae grouped in a sieve plate is characteristic for mid-lobular or centrilobular LSECs.

**Supporting Figure S3. Full unedited blot for Fig 5.** Unprocessed, uncropped original scan of immunoblot of pro-HB-EGF from lysates of LSECs from young and aged rats. Rectangle indicates portion of image used in Figure 5.

 **Supporting Fig S4. Full unedited blot for Fig 7.** Unprocessed, uncropped original scans of immunoblots from hepatocytes isolated from young rats and from aged rats treated daily with either solvent control or two weeks of cinaciguat. Rectangles indicate portion of image used in Fig 7.

**REFERENCES- supporting information**

[1]	DeLeve L, Wang X, Hu L, et al. Rat liver sinusoidal endothelial cell phenotype is maintained by paracrine and autocrine regulation. American Journal of Physiology-Gastrointestinal and Liver Physiology. 2004;287:G757–G763.

[2]	Meredith MJ. Rat hepatocytes prepared without collagenase: prolonged retention of differentiated characteristics in culture. Cell Biol Toxicol. 1988;4:405–425.

[3]	McLean AJ, Cogger VC, Chong GC, et al. Age-related pseudocapillarization of the human liver. Journal of Pathology. 2003;200:112–117.
